# Does turning trauma patients with an unstable spinal injury from the supine to a lateral position increase the risk of neurological deterioration? – A systematic review

**DOI:** 10.1186/s13049-015-0143-x

**Published:** 2015-09-17

**Authors:** Per Kristian Hyldmo, Gunn E. Vist, Anders Christian Feyling, Leif Rognås, Vidar Magnusson, Mårten Sandberg, Eldar Søreide

**Affiliations:** Norwegian Air Ambulance Foundation, Department of Research and Development, Drøbak, Norway; Department of Anesthesiology and Intensive Care, Sørlandet Hospital, Kristiansand, Norway; The Norwegian Knowledge Center for the Health Services, Oslo, Norway; Department of Anesthesiology, Oslo University Hospital, Oslo, Norway; Pre-hospital Critical Care Services, Aarhus, Denmark; Department of Anesthesiology, Landspitalinn University Hospital, Reykjavík, Iceland; Faculty of Medicine, University of Oslo, Oslo, Norway; Air Ambulance Department, Oslo University Hospital, Oslo, Norway; Network for Medical Sciences, University of Stavanger, Stavanger, Norway; Department of Anesthesiology and Intensive Care, Stavanger University Hospital, Stavanger, Norway

## Abstract

**Background:**

Airway protection and spinal precautions are competing concerns in the treatment of unconscious trauma patients. The placement of such patients in a lateral position may facilitate the acquisition of an adequate airway. However, trauma dogma dictates that patients should be transported in the supine position to minimize spinal movement. In this systematic review, we sought to answer the following question: Given an existing spinal injury, will changing a patient’s position from supine to lateral increase the risk of neurological deterioration?

**Methods:**

The review protocol was published in the PROSPERO database (Reg. no. CRD42012001190). We performed literature searches in PubMed, Medline, EMBASE, the Cochrane Library, CINAHL and the British Nursing Index and included studies of traumatic spinal injury, lateral positioning and neurological deterioration. The search was updated prior to submission. Two researchers independently completed each step in the review process.

**Results:**

We identified 1,164 publications. However, none of these publications reported mortality or neurological deterioration with lateral positioning as an outcome measure. Twelve studies used movement of the injured spine with lateral positioning as an outcome measure; eleven of these investigations were cadaver studies. All of these cadaver studies reported spinal movement during lateral positioning. The only identified human study included eighteen patients with thoracic or lumbar spinal fractures; according to the study authors, the logrolling technique did not result in any neurological deterioration among these patients.

**Conclusions:**

We identified no clinical studies demonstrating that rotating trauma patients from the supine position to a lateral position affects mortality or causes neurological deterioration. However, in various cadaver models, this type of rotation did produce statistically significant displacements of the injured spine. The clinical significance of these cadaver-based observations remains unclear. The present evidence for harm in rotating trauma patients from the supine position to a lateral position, including the logroll maneuver, is inconclusive.

**Electronic supplementary material:**

The online version of this article (doi:10.1186/s13049-015-0143-x) contains supplementary material, which is available to authorized users.

## Background

**Fig. 1:**
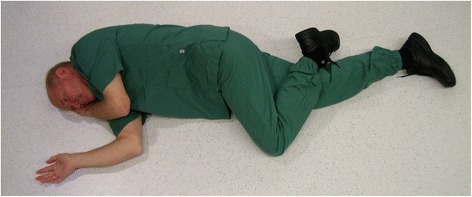
Recovery position

According to international resuscitation guidelines airway protection takes priority over spinal protection [1, 2]. This prioritization means that unconscious patients should be turned from the supine to the lateral position (“recovery position”) to maintain an open airway (Fig. 1). However, in trauma patients, this recommendation results in a dilemma for the basic emergency care provider. While the recovery position may be preferable for maintaining an open airway, existing dogma in traumatology dictates strict spinal immobilization in the supine position to minimize any spinal movement. For this reason, spinal precautions are an integral part of most trauma treatment [3, 4]. The fear of medical litigation may also be a factor in the development of guidelines for prehospital emergency care of unconscious trauma patients.

Kwan *et al.* have raised the question of whether spinal immobilization may actually endanger the airway in unconscious trauma patients [5]. Furthermore, some authors state that spinal precautions do not make a difference in morbidity [6, 7], should not be routinely used [8] and may even increase mortality [9].

For advanced prehospital providers, endotracheal intubation (ETI) has been considered the method of choice to secure the airway in unconscious trauma patients. However, some investigators have raised the question whether prehospital ETI actually reduces mortality and morbidity in trauma patients [10–12]. Further, on a global scale few emergency medical services (EMS) have personnel adequately trained in trauma ETI. ETI has been linked to the neurological exacerbation of an existing cervical spine injury [13].

**Fig. 2:**
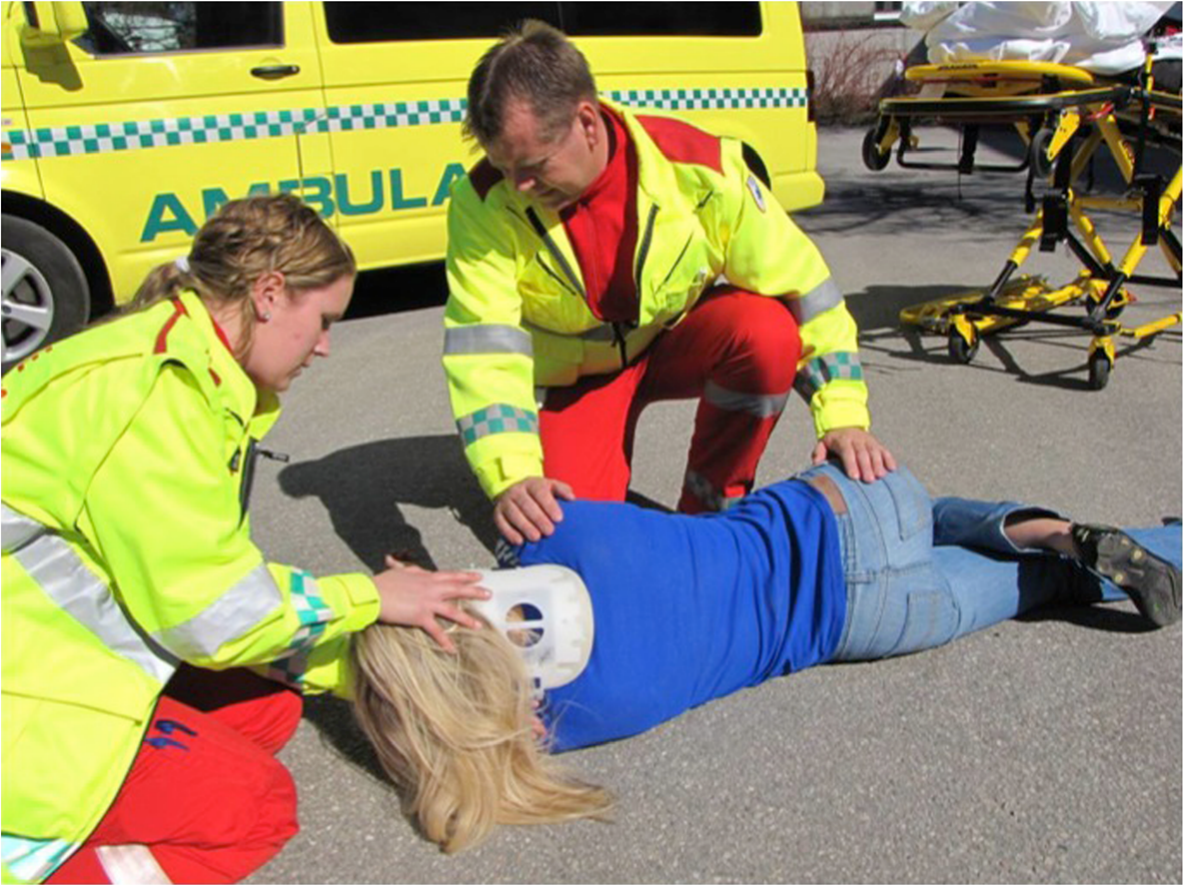
The Lateral Trauma Position. At least two rescuers are turning the patient in a modified logroll, maintaining a neutral axis of the spine

**Fig. 3:**
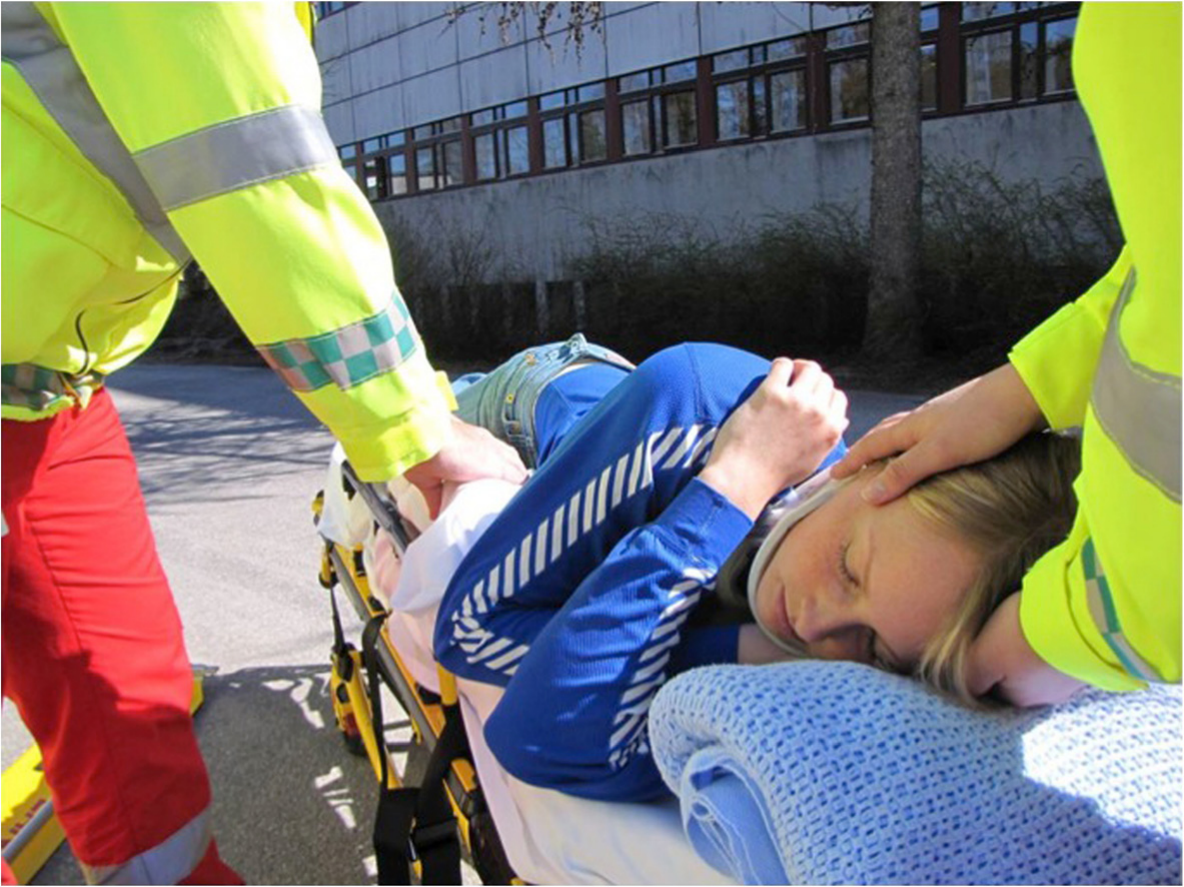
The Lateral Trauma Position on an ambulance gurney. The patient will be secured with the ambulance gurney straps. In this position the head can be manually stabilized, and the patient ventilated by bag-valve-mask. The addition of a vacuum mattress would further restrict spinal movement

To address this therapeutic dilemma in the prehospital setting, the Lateral Trauma Position (LTP) (Figs. 2 and 3) has been recommended [14] and implemented in some EMS systems [15]. One fundamental assumption regarding the LTP is that the extensively used logroll maneuver [3, 4] (i.e., rolling the supine patient sideways like a log, striving to maintaining a neutral axis of the spine) in unconscious trauma patients is safe.

It is still unclear whether the recommendation to immobilize unconscious trauma patients in the supine position is based on dogma alone or whether any supporting scientific evidence exists. We therefore performed a systematic review of the literature, using the following two questions. 1) In the unconscious trauma patient, is the supine position associated with a loss of airway patency compared with the lateral position? 2) Given an existing spinal injury, will changing the position of the patient from supine to lateral increase the risk of neurological deterioration? We addressed the former question in a recent publication [16]. Not surprisingly, we found that the supine position was associated with airway compromise. In the present report we address the latter question.

## Methods

The protocol for this review was published in the PROSPERO database for systematic reviews [17]. We used the PICO (Population, Intervention, Comparison and Outcome measures) format to develop the research question and the search strategies [18]. To ensure the quality of the process, we used the PRISMA (Preferred Reporting Items for Systematic Reviews and Meta-Analyses) checklist [19].

### Search and inclusion

#### Search methods for identification of studies

We searched the following databases: PubMed, MedLine, EMBASE, the Cochrane Library, CINAHL and the British Nursing Index. We modified our search terms as necessary when searching different databases. Citation searches were performed, and the “gray” literature (such as relevant textbooks) was searched manually. We applied no limits on the publication date. We did not use any language restrictions in the search; publications in languages other than English, German and Nordic languages were considered for translation. The MEDLINE search strategy is shown in Table 1. The complete search strategy can be found in Additional file 1.

**Table 1:** The MEDLINE search strategy

1	Spinal Cord Injuries/
2	Exp Back Injuries/
3	cervical vertebrae/in or lumbar vertebrae/in or thoracic vertebrae/in
4	(myelopath* adj2 (trauma* or post-trauma*)).tw.
5	((spinal or spine* or back) adj2 (contusion* or injury or injuries or trauma* or laceration* or transection*)).tw.
6	cervical vertebrae/ or lumbar vertebrae/ or thoracic vertebrae/
7	((cervical or lumbar or lumbalis or thoracic or thoracal or thoracolumbar or neck or cervicodorsal) adj2 (spine or spinal or backbone or column or vertebra* or canal)).tw.
8	“Wounds and Injuries”/
9	(wound* or injur* or trauma*).tw.
10	(6 or 7) and (8 or 9)
11	1 or 2 or 3 or 4 or 5 or 10
12	Patient positioning/
13	(patient* adj2 position*).tw.
14	Transportation of patients/
15	(patient* adj2 (transport* or maneuver* or moving or transfer*)).tw.
16	Exp Immobilization/
17	Immobili?ation*.tw.
18	((recovery or lateral) adj2 (posture* or position*)).tw.
19	ltp.tw.
20	Log roll*.tw.
21	Haines*.tw.
22	(high adj arm*).tw.
23	atls.tw.
24	phtls.tw.
25	or/12-24
26	11 and 25
27	Trauma severity indices/ or injury severity score/
28	“Severity of Illness Index”/
29	((rating or asia or injur* or trauma*) adj2 (score* or scale or severit*)).tw.
30	Motion/ or rotation/
31	Range of Motion, Articular/
32	(rotation or motion or (axis adj chang*) or (translat* adj4 (lateral or ap))).tw.
33	or/27-32
34	26 and 33

#### Types of participants

Due to the expected paucity of studies, we decided to include cadaver studies reporting movement in an existing spinal injury during positioning.

#### Types of interventions

We defined turning a person or cadaver into any lateral position as the intervention.

#### Types of outcome measures

We intended to use mortality rate as an outcome measure, along with any outcome measures related to neurological function. However, due to the expected lack of studies reporting these outcomes, we also included more indirect outcome measures such as angulation and translation (linear movement) in unstable spine injuries.

#### Types of studies

Due to the expected paucity of studies and the relatively low incidence of unstable spine injuries, we included all study designs, including case reports. Studies that included a control or comparison group formed the basis for our analysis and conclusions regarding the effect of the interventions.

### Data collection and analysis

The principal investigator (PKH) assessed all titles, abstracts and full-text articles identified in the searches. The remaining authors assessed one section each, ensuring that two investigators independently assessed each reference. We resolved any disagreement through discussion or, when required, consulted one of the other authors.

### Data extraction and management

For eligible studies, two authors of the review independently extracted the data using a data extraction form. We extracted data on first author, year of publication, population, details of the intervention and comparisons, outcome measures, measurement method and results.

### Assessment of risk of bias in included studies

Two review authors independently assessed the risk of bias for each study, using the criteria outlined in the Cochrane Handbook for Systematic Reviews of Interventions [20] or the checklists from the Norwegian Knowledge Centre for the Health Services [21]. We resolved any disagreements by discussion or by involving a third assessor.

The risk of bias assessment involved the following domains: sequence generation; allocation concealment; blinding of participants, providers and assessors; and incomplete outcome data, including possible attrition bias and selective reporting bias.

### Measures of treatment effect

#### Dichotomous data

For dichotomous data, we planned to present the results as summary risk ratios (RR) with 95 % confidence intervals (CI). No studies reporting dichotomous data were found.

#### Continuous data

For continuous data, we used the mean differences with standard deviations when outcomes were measured in the same way between trials. We have reported the median values of the means found in the different studies and the range of these means.

### Analysis

We have presented the results from the different studies in tables; it would not have been appropriate to conduct a meta-analysis.

### Missing data

All included studies were crossover studies that used the patients or cadavers as their own controls; there was no attrition in these studies. For continuous measures, we used actual measurements (no imputations).

### Assessment of heterogeneity

If applicable and sufficient data were identified, we planned to examine the meta-analysis forest plot for heterogeneity among the studies.

### Grading the quality of the evidence

We planned to use the GRADE methodology [22] for patient-critical and patient-important outcome measures, but as we identified only very indirect outcome measures, we were unable to do so.

## Results

We did not identify any randomized controlled trials (RCTs), observational studies or case reports that used mortality or neurological deterioration due to change from the supine to lateral position as outcome measures.

**Fig. 4:**
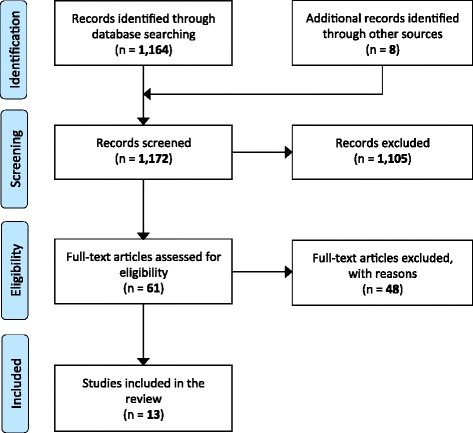
Inclusion and exclusion of studies, reading: "A list of the 48 excluded full-text articles, with reasons for exclusion can be found in Additional file 2"

Of the thirteen studies identified, eleven [23–33] were cadaver studies reporting movement in an artificially created unstable spinal injury model, e.g., during the logroll maneuver. We judged the studies to be well conducted. Figure 4 shows the inclusion and exclusion of studies.

Eight of these studies [23–30] reported angulation in cervical injuries (Table 2), three [26–28] reported translation (linear movement) in cervical injuries (Table 3), and three [31, 32, 34] reported angulation in thoraco-lumbar injuries (Table 4). Some of the investigators found considerable movement in globally unstable injuries, e.g., during logroll, especially in the lower thoracic and upper lumbar segments.

**Table 2:** Cervical angulation during logroll

Outcome	Study	Mean (SD)	Median	Range of means	
Flexion/extension	8 studies		5.5	2.8 - 11.9	
(Degrees)	Conrad 2007 [23]	7.3 (5.8)			
	Del Rossi 2004 [24]	2.8 (1.5)			
		3.1 (0.6)			
		3.4 (0.8)			
	Del Rossi 2004 [25]	3.9 (2.4)			
	Del Rossi 2008 [26]	5.5 (0.6)			
	Del Rossi 2010 [27]	6.0			
	Horodyski 2011 [28]	3.6 (2.3)			
	Prasarn 2012 [29]	11.9 (5.0)			
		9.1 (1.9)			
	Rechtine 2007 [30]	6.7			
Lateral bending	7 studies		4.0	1.6 - 9.2	
(Degrees)	Conrad 2007 [23]	9.2 (7.2)			
	Del Rossi 2004 [24]	2.3 (1.1)			
		1.6 (0.7)			
		1.8 (0.7)			
	Del Rossi 2008 [26]	7.5 (0.7)			
	Del Rossi 2010 [27]	3.8			
	Horodyski 2011 [28]	3.3 (1.7)			
	Prasarn 2012 [29]	8.7 (1.9)			
		6.7 (1.8)			
	Rechtine 2007 [30]	4.1			
Axial rotation	7 studies		6.3	2.4 - 7.9	
(Degrees)	Conrad 2007 [23]	7.9 (9.1)			
	Del Rossi 2004 [24]	6.1 (1.1)			
		6.4 (1.4)			
		7.1 (1.3)			
	Del Rossi 2008 [26]	7.2 (0.7)			
	Del Rossi 2010 [27]	3.1			
	Horodyski 2011 [28]	3.3 (1.9)			
	Prasarn 2012 [29]	7.5 (2.7)			
		5.8 (3.2)			
	Rechtine 2007 [30]	2.4			

Shows data from cadaver studies with artificially induced unstable cervical spine injuries. The standard deviation is shown for the studies in which it was reported. We report the medians of the means reported in the different studies and the range of these means. Del Rossi (2004) reported data from three different cervical collars, and Prasarn (2012) reported data for logrolls onto and off a backboard

**Table 3:** Cervical translation (linear movement) during logroll

Outcome	Study	Mean (SD)	Median	Range of means	
Anterior/posterior	3 studies		4.1	3.9 - 4.1	
(mm)	Del Rossi 2008 [26]	4.1 (0.6)			
	Del Rossi 2010 [27]	4.1			
	Horodyski 2011 [28]	3.9 (2.9)			
Axial	3 studies		4.1	2.6 - 4.9	
(mm)	Del Rossi 2008 [26]	4.9 (0.7)			
	Del Rossi 2010 [27]	4.1			
	Horodyski 2011 [28]	2.6 (1.8)			
Medial/lateral	3 studies		4.8	3.1 - 6.3	
(mm)	Del Rossi 2008 [26]	6.3 (0.6)			
	Del Rossi 2010 [27]	4.8			
	Horodyski 2011 [28]	3.1 (1.9)			

Shows data from cadaver studies with artificially induced unstable cervical spine injuries. The standard deviation is shown for the studies in which it was reported. We report the medians of the means reported in the different studies and the range of these means

**Table 4:** Thoracolumbar angulation during logroll

Outcome	Study	Mean	Median	Range of means	
Flexion/extension	3 studies		10.6	7.8 - 18.3	
(degrees)	Rubery 2013 [31]	8.6			
	Prasarn 2012 [32]	12.6			
		18.3			
	Del Rossi 2008b [34]	7.8			
Lateral bending	3 studies		8.6	6.9 - 10.5	
(degrees)	Rubery 2013 [31]	6.9			
	Prasarn 2012 [32]	10.1			
		10.5			
	Del Rossi 2008b [34]	7.0			
Axial rotation	3 studies		15.8	10.2 - 25.2	
(degrees)	Rubery 2013 [31]	13.7			
	Prasarn 2012 [32]	25.2			
		17.7			
	Del Rossi 2008b [34]	10.2			

Shows data from cadaver studies with artificially created unstable thoraco-lumbar spine injuries. The standard deviation is shown for the studies in which it was reported. We report the medians of the means reported in the different studies and the range of these means. Prasarn (2012) reports data of logrolls onto and off a backboard

The only human study [35] reported on eighteen patients with thoracic or lumbar spinal fractures. The outcome measure was the proportion of patients with “significant displacement” in the fracture when turning the patient from the supine to a lateral position in a controlled manner (“logroll maneuver”). “Significant displacement” was defined as more than a 3-mm linear movement and more than a 5° angulation in the planes studied. The proportion of patients with “significant displacement” varied from 0/18 to 6/18 in the various planes. However, according to the authors, “none of the patients suffered any neurological deficit as a result of the logrolling technique.” [35]

A 1987 publication reported thoracolumbar movement during logrolls in a healthy volunteer, a fresh cadaver with a surgically induced spinal lesion, and a patient with a Th12-L1 fracture [36]. They found substantial displacement during the logrolls, but they did not report any neurological deterioration in the patient.

## Discussion

We did not identify any interventional or observational clinical studies reporting neurological outcomes related to turning trauma patients from the supine to the lateral position. Furthermore, we did not identify any published case reports on this subject. However, we identified published case reports on neurological deterioration during airway management in patients with spinal injuries [13].

The only publications identified that addressed the potential secondary worsening of spinal neurological injury in general were of a historical character and may have led to the worldwide use of spinal precautions. In 1957, Rogers stated that [37], “It is a sad commentary that in one in every 10 patients, symptoms of cord compression or an increase in cord symptoms develop subsequent to the time of original injury - during emergency care, during the time when the diagnosis was being established, during definitive treatment or following reduction.” Rogers did not, however, attribute the development of these symptoms to any specific events during the phases of care that he described. In 1966, Geisler *et al.* described a study of 958 trauma patients with a spinal injury, 29 of whom had a delayed onset of symptoms [38]. The authors stated that “The paralysis occurred in each case as a consequence of failure to recognize the injury to the spinal column and to protect the patient from the consequences of his unstable spine.” In a 1988 study, Toscano reported that 26 % (32/124) of patients with significant spinal injury had major neurological deterioration between the time of injury and arrival at a spinal care unit [39]. This result has been interpreted to favor rigid spinal precaution protocols. However, Toscano stated in this report that “…it can be difficult to ascertain how much deterioration was due to the ‘natural disease process’ and how much deterioration was due to inappropriate handling.” He further noted that “…it is impossible to determine whether neurological deterioration is due to spinal cord oedema, a vascular problem, or inappropriate handling of the patient as the patient’s neurological deterioration developed over a period of time.” [39]

In our opinion, a variety of causes, including slowly progressive edema, hematoma, or a loss of tissue oxygenation and perfusion (as observed in other parts of the central nervous system) [40], may have contributed to the aforementioned cases. However, it may be difficult to identify the primary cause of neurologic deterioration in such instances.

The dogma of spinal immobilization seems to have originated in the 1960s and ’70s, apparently without much scientific evidence. This dogma has not been verified by clinical studies and generally has not been challenged since. The 1998 findings of Hauswald *et al.* indicate that strict spinal immobilization is not superior to no immobilization [6]. Furthermore, in a 2012 report, Hauswald argued that the deposition of energy into the spine is far greater during the injury phase than during post-injury handling [7]. In contrast, in most of the cases examined by Todd et al. in a recent publication, the cause of secondary neurological deterioration was thought to be failure to immobilize or the untimely removal of immobilization [41]. These results demonstrate that there remains considerable uncertainty regarding the general role of spinal immobilization.

In the current systematic review, we specifically searched for publications that reported neurological deterioration after trauma patients were rotated from the supine position to a lateral position. There was a dearth of such clinical studies. We therefore decided to expand the inclusion criteria to include studies that reported movement of the injured spine during lateral positioning. All of the cadaver studies that we reviewed described statistically significant displacements in artificially induced spinal injuries during lateral positioning. However, it is unclear whether these displacements are clinically significant. We have found no reports describing specific quantities of movement that represent thresholds for spinal cord damage and consequent neurological deterioration.

One possible limitation of our systematic review is that we searched very specifically for publications that studied the effect of turning a trauma patient from the supine to the lateral position. With a broader search, we may have identified other studies more indirectly relevant to the specific question. Nevertheless, we performed an extensive search with liberal inclusion criteria; we also searched the references of identified publications. Hence, it seems reasonable to conclude that we only identified a few studies due to a true lack of data on this subject.

Another possible limitation is that health care providers may be reluctant to report adverse outcomes. However, it seems unlikely that major neurological deterioration linked to turning of patients has been witnessed without any case reports being published.

Cadaver studies, such as those included in the present review, have been criticized for being performed on tissue that is not comparable to living tissue and for being based on overly unstable injuries. Regarding the latter, we see these injuries as a worst-case scenario, and the results are valuable as such. A major concern, however, is the lack of reliable means to correlate movement in the cadaver models to neurological outcomes in live patients.

The apparent lack of relevant studies does not necessarily imply that patients cannot be harmed by lateral positioning. Instead, it appears that relevant clinical studies have not yet been performed. A prospective, randomized controlled trial comparing standard supine immobilization to the lateral positioning of unconscious patients may not be feasible for logistical and ethical reasons. However, a prospective multi-center observational study with sufficient patients, similar to Hauswald’s 1998 investigation [6], may be a feasible alternative approach [42, 43].

Based on balancing the demonstrated risk of airway compromise in the supine position with the potential risk of secondary neurological deterioration when rotating a patient into a lateral position, it may be deemed acceptable to rotate unconscious trauma patients into a lateral position while simultaneously attempting to restrict spinal movement [15].

## Conclusions

In this systematic review, we identified no clinical studies demonstrating that rotating trauma patients from the supine position to a lateral position affects mortality or causes neurological deterioration. However, in various cadaver models, this type of rotation did produce statistically significant displacements of the injured spine. The clinical significance of these cadaver-based observations remains unclear. The present evidence for harm in rotating trauma patients from the supine position to a lateral position, including the logroll maneuver, is inconclusive.

### Consent

Written informed consent was obtained from the models for publication of the accompanying images.

## Additional files

Additional file 1:
**Search strategy.** (DOC 111 kb) Additional file 2:
**Excluded studies with reasons.** (DOCX 45 kb)
