# Supplementary material for: Does turning trauma patients with an unstable spinal injury from the supine to a lateral position increase the risk of neurological deterioration? – A systematic review
Source: Scand J Trauma Resusc Emerg Med. 2015 Sep 17;23:65. doi: 10.1186/s13049-015-0143-x (PMC4573694; doi:10.1186/s13049-015-0143-x)
Supplement: Additional file 1: — Search strategy. (DOC 111 kb) [file 13049_2015_143_MOESM1_ESM.doc]

# Search strategy

*Search completed 27.01.2012, updated 02.07.2014*

***PICO question***

*Given an existing spinal (cord) injury, will changing the patient’s position from supine to lateral (as in lateral/recovery position or log roll) worsen the spinal injury?*

**Database: Ovid MEDLINE**

| 1 | Spinal Cord Injuries/ (24450) |
| --- | --- |
| 2 | exp Back Injuries/ (16744) |
| 3 | cervical vertebrae/in or lumbar vertebrae/in or thoracic vertebrae/in (8957) |
| 4 | (myelopath* adj2 (trauma* or post-trauma*)).tw. (79) |
| 5 | ((spinal or spine* or back) adj2 (contusion* or injury or injuries or trauma* or laceration* or transection*)).tw. (26300) |
| 6 | cervical vertebrae/ or lumbar vertebrae/ or thoracic vertebrae/ (63177) |
| 7 | ((cervical or lumbar or lumbalis or thoracic or thoracal or thoracolumbar or neck or cervicodorsal) adj2 (spine or spinal or backbone or column or vertebra* or canal)).tw. (54006) |
| 8 | "Wounds and Injuries"/ (56509) |
| 9 | (wound* or injur* or trauma*).tw. (636986) |
| 10 | (6 or 7) and (8 or 9) (15857) |
| 11 | 1 or 2 or 3 or 4 or 5 or 10 (55125) |
| 12 | patient positioning/ (692) |
| 13 | (patient* adj2 position*).tw. (4627) |
| 14 | transportation of patients/ (7563) |
| 15 | (patient* adj2 (transport* or maneuver* or moving or transfer*)).tw. (8051) |
| 16 | exp Immobilization/ (21028) |
| 17 | immobili?ation*.tw. (27326) |
| 18 | ((recovery or lateral) adj2 (posture* or position*)).tw. (3642) |
| 19 | ltp.tw. (6454) |
| 20 | log roll*.tw. (39) |
| 21 | haines*.tw. (63) |
| 22 | (high adj arm*).tw. (43) |
| 23 | atls.tw. (415) |
| 24 | phtls.tw. (20) |
| 25 | or/12-24 (72164) |
| 26 | 11 and 25 (1833) |
| 27 | trauma severity indices/ or injury severity score/ (13401) |
| 28 | "Severity of Illness Index"/ (136130) |
| 29 | ((rating or asia or injur* or trauma*) adj2 (score* or scale or severit*)).tw. (37097) |
| 30 | motion/ or rotation/ (26948) |
| 31 | Range of Motion, Articular/ (27516) |
| 32 | (rotation or motion or (axis adj chang*) or (translat* adj4 (lateral or ap))).tw. (122524) |
| 33 | or/27-32 (324324) |
| 34 | 26 and 33 (274) |

**Database: Embase**

| 1 | exp spine injury/ (29718) |
| --- | --- |
| 2 | exp spinal cord injury/ (43230) |
| 3 | ((spinal or spine* or back) adj2 (contusion* or injury or injuries or trauma* or laceration* or transection*)).tw. (32088) |
| 4 | (myelopath* adj2 (trauma or post-trauma*)).tw. (31) |
| 5 | or/1-4 (76050) |
| 6 | cervical spine/ or lumbar spine/ or thoracic spine/ or thoracolumbar spine/ (51822) |
| 7 | ((cervical or lumbar or lumbalis or thoracic or thoracal or thoracolumbar or neck or cervicodorsal) adj2 (spine or spinal or backbone or column or vertebra* or canal)).tw. (65283) |
| 8 | injury/ (209008) |
| 9 | (wound* or injur* or trauma*).tw. (770699) |
| 10 | (6 or 7) and (8 or 9) (18040) |
| 11 | 5 or 10 (82838) |
| 12 | spine instabilitly/ or spine stabilization/ (5149) |
| 13 | patient positioning/ (10632) |
| 14 | (patient* adj2 position*).tw. (6186) |
| 15 | patient transport/ (16302) |
| 16 | (patient* adj2 (transport* or maneuver* or moving or transfer*)).tw. (10581) |
| 17 | immobilization/ (26904) |
| 18 | Immobili?ation*.tw. (34017) |
| 19 | ((recovery or lateral) adj2 (posture* or position*)).tw. (4493) |
| 20 | ltp.tw. (7880) |
| 21 | log roll*.tw. (47) |
| 22 | haines*.tw. (74) |
| 23 | (high adj arm*).tw. (56) |
| 24 | atls.tw. (515) |
| 25 | phtls.tw. (22) |
| 26 | or/12-25 (106855) |
| 27 | 11 and 26 (4858) |
| 28 | rating scale/ (71028) |
| 29 | injury scale/ (10147) |
| 30 | ((rating or asia or injur* or trauma*) adj2 (score* or scale or severit*)).tw. (47749) |
| 31 | motion/ or rotation/ (27945) |
| 32 | motion analysis system/ (1507) |
| 33 | range of motion/ (10276) |
| 34 | (rotation or motion or (axis adj change) or (translation adj4 (lateral or ap))).tw. (147976) |
| 35 | or/28-34 (272027) |
| 36 | 27 and 35 (634) |

**Database: Cochrane Library**

| #1 | MeSH descriptor Spinal Cord Injuries explode all trees | 747 |
| --- | --- | --- |
| #2 | MeSH descriptor Back Injuries explode all trees | 636 |
| #3 | (myelopath* NEAR/3 (trauma* or post-trauma*)):ti,ab,kw | 2 |
| #4 | ((spinal or spine* or back) NEAR/3 (contusion* or injury or injuries or trauma* or laceration* or transection*)):ti,ab,kw | 1260 |
| #5 | (#1 OR #2 OR #3 OR #4) | 1818 |
| #6 | MeSH descriptor Cervical Vertebrae explode all trees | 592 |
| #7 | MeSH descriptor Lumbar Vertebrae explode all trees | 1693 |
| #8 | MeSH descriptor Thoracic Vertebrae explode all trees | 247 |
| #9 | ((cervical or lumbar or lumbalis or thoracic or thoracal or thoracolumbar or neck or cervicodorsal) NEAR/3 (spine or spinal or backbone or column or vertebra* or canal)):ti,ab,kw | 4706 |
| #10 | (#6 OR #7 OR #8 OR #9) | 4710 |
| #11 | MeSH descriptor Wounds and Injuries explode all trees | 12843 |
| #12 | (wound* or injur* or trauma*):ti,ab,kw | 30680 |
| #13 | (#11 OR #12) | 34943 |
| #14 | (#10 AND #13) | 913 |
| #15 | (#5 OR #14) | 2275 |
| #16 | MeSH descriptor Patient Positioning explode all trees | 39 |
| #17 | (patient* NEAR/2 position*):ti,ab,kw | 449 |
| #18 | MeSH descriptor Transportation of Patients explode all trees | 172 |
| #19 | (patient* NEAR/3 (transport* or maneuver* or moving or transfer*)):ti,ab,kw 880 |  |
| #20 | MeSH descriptor Immobilization explode all trees | 523 |
| #21 | immobili?ation*:ti,ab,kw | 947 |
| #22 | ((recovery or lateral) NEAR/3 (posture* or position*)):ti,ab,kw | 523 |
| #23 | (ltp or haines* or atls or phtls):ti,ab,kw | 61 |
| #24 | (log roll*):ti,ab,kw | 8 |
| #25 | (high NEAR/2 arm*):ti,ab,kw | 235 |
| #26 | (#16 OR #17 OR #18 OR #19 OR #20 OR #21 OR #22 OR #23 OR #24 OR #25) 3181 |  |
| #27 | (#15 AND #26) | 98 |

**Database: ISI Web of Science**

| # 16 | 209 | #15 AND #12 |
| --- | --- | --- |
| # 15 | 578312 | #14 OR #13 |
| # 14 | 519117 | TI=(rotation or motion or (axis adj change) or (translation adj4 (lateral or ap))) OR TS=(rotation or motion or (axis adj change) or (translation adj4 (lateral or ap))) |
| # 13 | 60565 | TI=((rating or asia or injur* or trauma*) NEAR/2 (score* or scale or severit*)) OR TS=((rating or asia or injur* or trauma*) NEAR/2 (score* or scale or severit*)) |
| # 12 | 1052 | #11 AND #5 |
| # 11 | 83304 | #10 OR #9 OR #8 OR #7 OR #6 |
| # 10 | 10553 | TI=(ltp or log roll* or haines* or (high NEAR/2 arm) or atls or phtls) OR TS=(ltp or log roll* or haines* or (high NEAR/2 arm) or atls or phtls) |
| # 9 | 4136 | TI=((recovery or lateral) NEAR/2 (posture* or position*)) OR TS=((recovery or lateral) NEAR/2 (posture* or position*)) |
| # 8 | 53856 | TI=immobili?ation* OR TS=immobili?ation* |
| # 7 | 9833 | TI=(patient* NEAR/2 (transport* or maneuver* or moving or transfer*)) OR TS=(patient* NEAR/2 (transport* or maneuver* or moving or transfer*)) |
| # 6 | 5744 | TI=(patient* NEAR/2 position*) OR TS=(patient* NEAR/2 position*) |
| # 5 | 40112 | #1 or #2 or (#3 and #4) |
| # 4 | 645887 | TI=(wound* or injur* or trauma*) OR TS=(wound* or injur* or trauma*) |
| # 3 | 54212 | TI=((cervical or lumbar or lumbalis or thoracic or thoracal or thoracolumbar or neck or cervicodorsal) NEAR/2 (spine or spinal or backbone or column or vertebra* or canal)) OR TS=((cervical or lumbar or lumbalis or thoracic or thoracal or thoracolumbar or neck or cervicodorsal) NEAR/2 (spine or spinal or backbone or column or vertebra* or canal)) |
| # 2 | 34293 | TI=((spinal or spine* or back) NEAR/2 (contusion* or injury or injuries or trauma* or laceration* or transection*)) OR TS=((spinal or spine* or back) NEAR/2 (contusion* or injury or injuries or trauma* or laceration* or transection*)) |
| # 1 | 103 | TI=(myelopath* NEAR/2 (trauma* or post-trauma*)) OR TS=(myelopath* NEAR/2 (trauma* or post-trauma*)) |

**Database: Cinahl**

| S32 | S25 and S31 | 148 |
| --- | --- | --- |
| S31 | S26 or S27 or S28 or S29 or S30 | 58047 |
| S30 | TI ( (rotation or motion or (axis N2 chang*) or (translat* N4 (lateral or ap))) ) OR AB ( (rotation or motion or (axis N2 chang*) or (translat* N4 (lateral or ap))) ) | 15535 |
| S29 | (MH "Motion+") OR (MH "Motion Analysis Systems") OR (MH "Range of Motion") | 12707 |
| S28 | TI ( ((rating or asia or injur* or trauma*) N2 (score* or scale or severit*)) ) OR AB ( ((rating or asia or injur* or trauma*) N2 (score* or scale or severit*)) ) | 9474 |
| S27 | (MH "Severity of Illness") | 8391 |
| S26 | (MH "Trauma Severity Indices+") OR (MH "Severity of Injury") OR (MH "Severity of Illness Indices") | 21925 |
| S25 | S14 and S24 | 934 |
| S24 | S15 or S16 or S17 or S18 or S19 or S20 or S21 or S22 or S23 | 17642 |
| S23 | TI ( (ltp or log roll* or haines* or (high N2 arm) or atls or phtls) ) OR AB ( (ltp or log roll* or haines* or (high N2 arm) or atls or phtls) ) | 250 |
| S22 | TI ( ((recovery or lateral) N2 (posture* or position*)) ) OR AB ( ((recovery or lateral) N2 (posture* or position*)) ) | 445 |
| S21 | TI ( immobilization* or immobilisation* ) OR AB ( immobilization* or immobilisation* ) | 1551 |
| S20 | (MH "Immobilization") | 1627 |
| S19 | TI ( (patient* N2 (transport* or maneuver* or moving or transfer*)) ) OR AB ( (patient* N2 (transport* or maneuver* or moving or transfer*)) ) | 2824 |
| S18 | (MH "Transportation of Patients+") | 6035 |
| S17 | TI (patient* n2 position*) OR AB (patient* n2 position*) | 1213 |
| S16 | (MH "Lateral Position") | 34 |
| S15 | (MH "Patient Positioning+") | 5936 |
| S14 | S6 or S13 | 17480 |
| S13 | S11 and S12 | 4388 |
| S12 | S9 or S10 | 169683 |
| S11 | S7 or S8 | 12368 |
| S10 | TI ( (wound* or injur* or trauma*) ) OR AB ( (wound* or injur* or trauma*) ) | 105460 |
| S9 | (MH "Wounds and Injuries+") | 118168 |
| S8 | TI ( ((cervical or lumbar or lumbalis or thoracic or thoracal or thoracolumbar or neck or cervicodorsal) N2 (spine or spinal or backbone or column or vertebra* or canal)) ) OR AB ( ((cervical or lumbar or lumbalis or thoracic or thoracal or thoracolumbar or neck or cervicodorsal) N2 (spine or spinal or backbone or column or vertebra* or canal)) ) | 7264 |
| S7 | (MH "Cervical Vertebrae+") OR (MH "Thoracic Vertebrae") OR (MH "Lumbar Vertebrae") | 9165 |
| S6 | S1 or S2 or S3 or S4 or S5 | 15963 |
| S5 | TI ( ((spinal or spine* or back) N2 (contusion* or injury or injuries or trauma* or laceration* or transection*)) ) OR AB ( ((spinal or spine* or back) N2 (contusion* or injury or injuries or trauma* or laceration* or transection*)) ) | 9236 |
| S4 | TI ( (myelopath* N3 (trauma* or post-trauma*)) ) OR AB ( (myelopath* N3 (trauma* or post-trauma*)) ) | 15 |
| S3 | (MH "Cervical Vertebrae+/IN") OR (MH "Lumbar Vertebrae/IN") OR (MH "Thoracic Vertebrae/IN") | 1346 |
| S2 | (MH "Back Injuries+") | 3901 |
| S1 | (MH "Spinal Cord Injuries+") 10158 |  |
