# Supplementary material for: Does turning trauma patients with an unstable spinal injury from the supine to a lateral position increase the risk of neurological deterioration? – A systematic review
Source: Scand J Trauma Resusc Emerg Med. 2015 Sep 17;23:65. doi: 10.1186/s13049-015-0143-x (PMC4573694; doi:10.1186/s13049-015-0143-x)
Supplement: Additional file 2: — Excluded studies with reasons. (DOCX 45 kb) [file 13049_2015_143_MOESM2_ESM.docx]

## Excluded studies with reasons

| **Study** | **Reason for exclusion** |
| --- | --- |
| Ahn H, Singh J, Nathens A, MacDonald RD, Travers A, Tallon J, Fehlings MG, Yee A: **Pre-hospital care management of a potential spinal cord injured patient: a systematic review of the literature and evidence-based guidelines.** *J Neurotrauma* 2011, **28:**1341-1361. | Does not report data on lateral vs. supine position |
| Barker E, Saulino MF: **First-ever guidelines for spinal cord injuries.** *RN* 2002, **65:**32-37. | Review article, no original data, but we searched the references. |
| Bearden BG, Conrad BP, Horodyski M, Rechtine GR: **Motion in the unstable cervical spine: comparison of manual turning and use of the Jackson table in prone positioning.** *Journal of Neurosurgery-Spine* 2007, **7:**161-164. | Does not report data on lateral vs. supine position |
| Bernhard M, Gries A, Kremer P, Bottiger BW: **Spinal cord injury (SCI)--prehospital management.** *Resuscitation* 2005, **66:**127-139. | Healthy volunteers, i.e. no spinal fracture. |
| Blake WE, Stillman BC, Eizenberg N, Briggs C, McMeeken JM: **The position of the spine in the recovery position--an experimental comparison between the lateral recovery position and the modified HAINES position.** *Resuscitation* 2002, **53:**289-297. | Healthy volunteers, i.e. no spinal fracture. |
| Boissy P, Shrier I, Briere S, Mellete J, Fecteau L, Matheson GO, Garza D, Meeuwisse WH, Segal E, Boulay J, Steele RJ: **Effectiveness of cervical spine stabilization techniques.** *Clin J Sport Med* 2011, **21:**80-88. | Does not report data on lateral vs. supine position. Healthy volunteers, i.e. no spinal fracture. |
| Bonhomme V, Hans P: **Management of the unstable cervical spine: elective versus emergent cases.** *Current Opinion in Anesthesiology* 2009, **22:**579-585. | Review article, no original data, but we searched the references. |
| Conrad B, Horodyski MB, Del Rossi G, Prasarn M, Rechtine G, Vanderhave KL: **Cervical Spinal Trauma in Children and Adults: Perioperative Considerations.** *J Am Acad Orthop Surg* 2011, **19:**581-582. | Commentary article, no data. |
| Conrad BP, Marchese DL, Rechtine GR, Horodyski M: **Motion in the unstable thoracolumbar spine when spine boarding a prone patient.** *J Spinal Cord Med* 2012, **35:**53-57. | Does not report data on lateral vs. supine position |
| Dahl MC, Ananthakrishnan D, Nicandri G, Chapman JR, Ching RP: **Helmet and shoulder pad removal in football players with unstable cervical spine injuries.** *Journal of Applied Biomechanics* 2009, **APPL.:**119-132. | Model study, too indirect. |
| DelRossi G, Horodyski M, Powers ME: **A Comparison of Spine-Board Transfer Techniques and the Effect of Training on Performance.** *Journal of Athletic Training* 2003, **38:**204-208. | Healthy volunteers, i.e. no spinal fracture. |
| Dipaola CP, Conrad BP, Horodyski M, Dipaola MJ, Sawers A, Rechtine GR, 2nd: **Cervical spine motion generated with manual versus jackson table turning methods in a cadaveric c1-c2 global instability model.** *Spine (Phila Pa 1976)* 2009, **34:**2912-2918. | Does not report data on lateral vs. supine position |
| DiPaola CP, Dipaola MJ, Conrad BP, Horodyski M, Del Rossi G, Sawers A, Rechtine GR: **Comparison of thoracolumbar motion produced by manual and Jackson-table-turning methods. Study of a cadaveric instability model.** *Journal of Bone and Joint Surgery - American Volume* 2008, **90:**1698-1704. | Does not report data on lateral vs. supine position |
| Dipaola MJ, DiPaola CP, Conrad BP, Horodyski M, Del Rossi G, Sawers A, Bloch D, Rechtine GR: **Cervical spine motion in manual versus Jackson table turning methods in a cadaveric global instability model.** *Journal of Spinal Disorders and Techniques* 2008, **21:**273-280. | Does not report data on lateral vs. supine position |
| Ebraheim NA: **Biomechanical analysis of cervical and thoracolumbar spine motion in intact and partially and completely unstable cadaver spine models with kinetic bed therapy or traditional log roll: Commentary.** *Journal of Trauma - Injury, Infection and Critical Care* 2007, **62:**388. | Commentary article, no data. |
| Fellrath, Jr., Hanley, Jr.: **Multitrauma and thoracolumbar fractures.** *Seminars in Spine Surgery* 1995, **7:**103-108. | The only data reported are on timing of surgery. |
| Gunn BD, Eizenberg N, Silberstein M, McMeeken JM, Tully EA, Stillman BC, Brown DJ, Gutteridge GA: **How should an unconscious person with a suspected neck injury be positioned?** *PrehospDisasterMed* 1995, **10:**239-244. | Healthy volunteers, i.e. no spinal fracture. |
| Horodyski M, Weight M, Conrad B, Bearden B, Kimball J, Rechtine G: **Motion generated in the unstable lumbar spine during hospital bed transfers.** *J Spinal Disord Tech* 2009, **22:**45-48. | Does not report data on lateral vs. supine position |
| Hwang H, Hipp JA, Ben-Galim P, Reitman CA: **Threshold cervical range-of-motion necessary to detect abnormal intervertebral motion in cervical spine radiographs.** *Spine* 2008, **33:**E261-E267. | Does not report data on lateral vs. supine position |
| James KS, Wenger KH, Schlegel JD, Dunn HK: **Biomechanical evaluation of the stability of thoracolumbar burst fractures.** *Spine* 1994, **19:**1731-1740. | Does not report data on lateral vs. supine position. |
| Kwan I, Bunn F, Roberts IG: **Spinal immobilisation for trauma patients. Cochrane Database of Systematic Reviews: Reviews.** In *Cochrane Database of Systematic Reviews 2001 Issue 2.* Chichester (UK): John Wiley & Sons, Ltd; 2001 | Review article, no original data, but we searched the references. |
| Lador R, Ben-Galim P, Hipp JA: **Motion within the unstable cervical spine during patient maneuvering: the neck pivot-shift phenomenon.** *Journal of Trauma-Injury Infection and Critical Care* 2011, **70:**247-250. | Does not report data on lateral vs. supine position. |
| Linares HA, Mawson AR, Suarez E, Biundo JJ: **Association between pressure sores and immobilization in the immediate post-injury period.** *Orthopedics* 1987, **10:**571-573. | Outcome measure is pressure sores. |
| Mazolewski P, Manix TH: **The effectiveness of strapping techniques in spinal immobilization.** *Ann Emerg Med* 1994, **23:**1290-1295. | Healthy volunteers, i.e. no spinal fracture. |
| McCabe JB, Nolan DJ: **Comparison of the effectiveness of different cervical immobilization collars.** *Ann Emerg Med* 1986, **15:**50-53. | Healthy volunteers, i.e. no spinal fracture. |
| McGrath T, Murphy C: **Comparison of a SAM splint-molded cervical collar with a Philadelphia cervical collar.** *Wilderness Environ Med* 2009, **20:**166-168. | Healthy volunteers, i.e. no spinal fracture. |
| McGuire RA, Degnan G, Amundson GM: **Evaluation of current extrication orthoses in immobilization of the unstable cervical spine.** *Spine* 1990, **15:**1064-1067. | Does not report data on lateral vs. supine position |
| Rechtine GR, Del Rossi G, Conrad BP, Horodyski M: **Motion generated in the unstable spine during hospital bed transfers.** *The Journal of trauma* 2004, **57:**609-611; discussion 611-602. | Does not report data on lateral vs. supine position |
| Richter D, Latta LL, Milne EL, Varkarakis GM, Biedermann L, Ekkernkamp A, Ostermann PA: **The stabilizing effects of different orthoses in the intact and unstable upper cervical spine: a cadaver study.** *Journal of Trauma-Injury Infection and Critical Care* 2001, **50:**848-854. | Does not report data on lateral vs. supine position |
| Swartz EE, Nowak J, Shirley C, Decoster LC: **A comparison of head movement during back boarding by motorized spine-board and log-roll techniques.** *Journal of Athletic Training* 2005, **40:**162-168. | Healthy volunteers, i.e. no spinal fracture. |
| Vanderlan WB, Tew BE, Seguin CY, Mata MM, Yang JJ, Horst HM, Obeid FN, McSwain NE: **Neurologic sequelae of penetrating cervical trauma.** *Spine* 2009, **34:**2646-2653. | Does not report data on lateral vs. supine position |
| Yim M, Laucharoen J: **Towards Small Robot Aided Victim Manipulation.** *Journal of Intelligent & Robotic Systems* 2011, **64:**119-139. | Does not report data on lateral vs. supine position |
| Young WF, Shea M: **Acute management of spine and spinal cord injury.** *Trauma Quarterly* 1998, **14:**21-42. | Review article, no original data, but we searched the references. |
| Conrad BP, Rossi GD, Horodyski MB, Prasarn ML, Alemi Y, Rechtine GR: **Eliminating log rolling as a spine trauma order.** *Surg Neurol Int* 2012, **3:**S188-197. | Review article, no original data, but we searched the references. |
| Swartz EE, Del Rossi G: **Cervical spine alignment during on-field management of potential catastrophic spine injuries.** *Sports Health* 2009, **1:**247-252. | Review article, no original data, but we searched the references. |
| Del Rossi G, Rechtine GR, Conrad BP, Horodyski M: **Is sub-occipital padding necessary to maintain optimal alignment of the unstable spine in the prehospital setting? A preliminary report.** *J Emerg Med* 2013, **45:**366-371. | Does not report data on lateral vs. supine position |
| Hauswald M: **A re-conceptualisation of acute spinal care.** *Emergency Medicine Journal* 2013, **30:**720-723. | Review article, no original data, but we searched the references. |
| Ivancic PC: **Do cervical collars and cervicothoracic orthoses effectively stabilize the injured cervical spine? A biomechanical investigation.** *Spine* 2013, **38:**E767-774. | Model study, too indirect. |
| Hu CT, Dipaola CP, Conrad BP, Horodyski M, Del Rossi G, Rechtine GR: **Motion is reduced in the unstable spine with the use of mechanical devices for bed transfers.** *J Spinal Cord Med* 2013, **36:**58-65. | Does not report data on lateral vs. supine position |
| Leonard JC, Mao J, Jaffe DM: **Potential adverse effects of spinal immobilization in children.** *Prehosp Emerg Care* 2012, **16:**513-518. | Does not report data on lateral vs. supine position |
| Connor D, Greaves I, Porter K, Bloch M: **Pre-hospital spinal immobilisation: An initial consensus statement.** *Emergency Medicine Journal* 2013, **30:**1067-1069. | Guideline article, no data. |
| Horowitz MJ, Hughes TH, Mann FA: **Core Curriculum Illustration: Marked head rotation following blunt trauma.** *Emergency Radiology* 2013, **20:**163-164. | Does not report data on lateral vs. supine position |
| Stahel PF, Vanderheiden T, Finn MA: **Management strategies for acute spinal cord injury: Current options and future perspectives.** *Current Opinion in Critical Care* 2012, **18:**651-660. | Review article, no original data, but we searched the references. |
| Stein DM, Roddy V, Marx J, Smith WS, Weingart SD: **Emergency neurological life support: Traumatic spine injury.** *Neurocritical Care* 2012, **17:**S102-S111. | Review article, no original data, but we searched the references. |
| Mitra B, Cameron PA: **Optimising management of the elderly trauma patient.** *Injury* 2012, **43:**973-975. | Review article, no original data, but we searched the references. |
| CADTH: **The use of spine boards in the pre-hospital setting for the stabilization of patients following trauma: a review of the clinical evidence and guidelines.** *Health Technology Assessment Database* 2013. | Review article, no original data, but we searched the references. |
| National Association of EMS Physicians, American College of Surgeons Committee on Trauma: **EMS Spinal Precautions and the Use of the Long Backboard.** *Prehosp Emerg Care* 2013, **17:**392-393. | Guideline article, no data. |
| Bednar DA: **Efficacy of orthotic immobilization of the unstable subaxial cervical spine of the elderly patient: investigation in a cadaver model.** *Can J Surg* 2004, **47:**251-256. | Does not report data on turning from supine to lateral position. |
